# The Proposed Yellow Fever Commission and the National Board of Health

**Published:** 1886-05

**Authors:** 


### Correspondence.

### THE PROPOSED YELLOW FEVER COMMISSION AND THE NATIONAL BOARD OF HEALTH.

New Orleans, March 29, 1886.

*Editors Atlanta Medical and Surgical Journal*:

Gentlemen—From information just received, I learn that my letter of February 20th to Dr. J. C. LeHardy, Secretary Georgia Medical Society, cannot appear until May.

While regretting this, I fully understand the unavoidable nature of the delay.

If you will preface the article with a statement of the fact that the overcrowded condition of the March and April numbers prevented its earlier appearance, such an explanation would offset, in some measure, the question of lateness. However, the principles contained are as forcible and just in May as in February.

My entire defense has been in behalf of the autonomy of State health authorities, and in the resistance of an encroachment, not only unwarrantable in itself, but dangerous as a precedent, for the yielding of an inch would be the prelude to the surrender of an ell. We know with whom we are dealing.

In order to perfect the regulation of health matters in their relation to the country at large, and between the several States, there should be created under some department at Washington, we will say that of the Interior—a Bureau of Health in every particular analogous to the Bureau of Education, without patronage or public funds for disbursement, as set forth in a bill now before Congress. Above all, the health authorities in every State should be represented in and governed by the concensus of those great national organizations. "The American Public Health Association" (which is in fact continental, including Canada) and the Boards of Health of the United States.

Already the States of this valley are working in a perfect and harmonious obedience to the ordinances of the "Sanitary Council of the Mississippi Valley."

These institutions are thoroughly American and democratic in spirit, and their controlling influence wholesome and sufficient, while the National Board of Health is central, autocratic and insolently domineering.

It is more than useless to talk about fellowship with such a power, knowing that there can exist no relation, except one of defiant resistance or of truculent subserviency, which latter must imply the depravity and recreance of men who have been made the custodians of a sacred public trust. We are not prepared for its acceptance at such a price.

These are the reasons why "the *National Board was a premature birth*—born before the public had become enlightened in sanitation (*and truculency*) sufficienly either to comprehend its proper functions or to appreciate the great benefits which *an enlightened people* would force it to confer on them."

An undisguised eagerness for authority and inordinate grasping for money have too plainly stamped the character and history of the National Board to be mistaken by any American who values the methods and traditions of our republican system.

The States are not yet *sufficiently enlightened* to surrender to a centralized National Board the power to bind and to loosen. They are still in that state of primitive ignorance in which they maintain the principle that the people of a State are the best judges of their own internal necessities, and best able to determine police and other regulations growing out of them.

In the matter of public health preservation and the management of quarantine, the States can better agree upon a mutual arrangement than afford to commit such vital affairs, with the enormous power they confer upon any central board of nine men, itself to be wielded presently by the vast moneyed monopolies representing hostile commercial, railroad and steamship interests to tyrannize over and crush the weaker State or section.

The attempted forcible seizure of the Yellow Fever Commission was simply a desperate first step in the direction of regaining lost power and patronage.

I have resisted the National Board of Health, profoundly convinced that to do so was a righteous and patriotic resistance of the most mischievous and threatening organization to which the calamity of a people ever gave "*a premature birth.*"

Yours very truly, JOSEPH HOLT, M. D.

SAVANNAH, March 22, 1886.

*Editors Atlanta Medical and Surgical Journal:*

DEAR SIRS—I have received the enclosed letter from Dr. Joseph Holt, President Board of Health of Louisiana, with the request to have it published in a medical journal in Georgia. This letter being only part of a discussion which has been going on for some time in New Orleans, it would be unintelligible to some of your readers without an explanation of the events which led to its being written. When Dr. Holt was in Washington as a delegate from the American Public Health Association, and advocating before a committee of Congress the scheme proposed by himself, of sending a commission to South America and Mexico to study yellow fever, and especially the methods of Friere and Carmona of preventing it by inoculation, he was shown a letter from the resident member of the National Board of Health at New Orleans (Dr. Chaillé), detailing a plan for putting the proposed commission under the control of the National Board, and was approached by members of the same board resident in Washington, and urged to accede to this plan—being told that sending the commission under any other auspices would be a death-blow to the National Board. Naturally indignant at this attempt to appropriate to themselves the honor and credit of putting into effect a useful idea originating in some one else, Dr. Holt, on his return to New Orleans, published an "open letter" to Dr. Chaillé, in which he gave his opinion of the board and its methods. It was in reply to my inquiry for further information that the following letter was sent.

I think its publication at this time, when there is the prospect of a scientific bureau being established for sanitary purposes, will do much good in showing how the National Board of Health, after having been deprived of its functions by the deliberate judgment of Congress on charges of extravagance and uselessness, is striving to rehabilitate itself by surreptitious means. Should they succeed in this effort, we will be carried back to the old time of its active existence, when, through the recommendation of its Havana investigating committee, our Southern ports were sealed for six months of the year against alleged yellow fever latitudes· Then the exploded quarantine of detention was coaxed upon us at the South, in utter disregard of all the discoveries in the science of sanitation which had succceded in rendering infected ships from infected ports perfectly harmless, *provided they went north!*

Very respectfully yours,

J. C. LEHARDY, M. D.

OFFICE BOARD OF HEALTH, STATE OF LOUISIANA,
NEW ORLEANS, February 26, 1886.

*Dr. J. C. LeHardy, Savannah, Ga.:*

DEAR SIR—Your considerate letter of the 23rd instant has just been received. I endeavored to reply to your former note, but was interrupted by a pressure of work.

The whole question of the great injustice attempted by the National Board of Health was presented in a concise letter, written ostensibly to the resident member of the National Board, but really to the United States Senate. I felt assured that if Congress could have a true exhibition of the case, it would at once perceive how grossly unfair it would be to take the special business of a State Board of Health, endorsed and presented as a petition by the Boards of Health of the United States and the American Public Health Association, out of their hands, ignoring them utterly, and putting it into the keeping of an arrogant, self-assertive, close corporation, whose history is one of failure to achieve any good, but an unlimited ability to make enemies by compelling others to assume the defensive in order to resist the unwarrantable encroachments of an unbridled ambition.

In this particular instance, the National Board was free to have opened the question of sending into the tropics an investigating yellow fever commission.

Had it done so, the Louisiana State Board of Health would never have thrown in its way an obstruction of any kind, still less would it, or the American Public Health Association, have made an effort to displace the originators and ourselves coolly take possession.

The rule of justice works both ways. When this high-handed measure was tried on us, I resisted it as an outrageous piece of effrontery that should not be tolerated.

As the Senate Committee on Infectious Diseases had already expressed its willingness to commit the matter to the National Board of Health, it became necessary for me to address the members of the Senate Committee and of Congress generally, in order to prevent the doing unwittingly of an act of injustice.

The resident member of the National Board of Health furnished me the way by insisting upon an explanation of my remarks in a newspaper interview, after having been advised of my intention to send him in reply an open letter—for publication—if he still demanded an answer.

Thus it is our shrewdest enemies are sometimes of infinite service in working out retributive justice for our good.

After making himself so useful to our cause by helping us to a hearing, the lengthy reply of the resident member was delightful entertainment.

One of the conspicuous points in his address, "*To the Public*," published in the N. O. *Picayune*, 21st instant, is as follows: "The duties of the National Board of Health shall be to obtain information upon all mattern affecting the public health, and the members of this board shall make, or cause to be made, such special examinations and investigations at any place or places within the United States or foreign ports as they may deem best."

I immediately quoted this in telegrams to the chairmen of the two committees in the Senate and in the House, adding, "When Congress prescribed the functions of the National Board of Health it did not debar itself the right to confer the same privileges and to demand like service of any other agent it might choose." The entire conduct of the National Board of Health in neglecting to move in the important matter of the proposed investigation, but leaving it for others to suggest and plan, and finally to urge upon Congress, condemns it as a useless piece of national furniture unworthy of existence.

Its subsequent action in recognition of the importance of the investigation, as manifested by its eagerness to get possession of the commission, confirms the justness of this conclusion.

The confliet is one of resistance to usurpation. So far as we are concerned, the question is one of even-handed justice and of propriety, or rather of expediency.

Would it be proper to commit the work of the proposed commission to a body whose representative has repeatedly expressed an opinion of its uselessness ? Good results can only be expected from those who are enthusiastically in earnest and full of hope.

From every consideration of right, and desire to accomplish a great public good, the commission should be kept in the hands of its natural parents, and not be farmed out to find nourishment at the withered breasts of a poverty-stricken wet-nurse.

Yours very truly,

(Signed) JOSEPH HOLT, M. D.,

President Board of Health, State of Louisiana.

